# Migraine and Irritable Bowel Syndrome Among the General Population in Aseer Region

**DOI:** 10.7759/cureus.45047

**Published:** 2023-09-11

**Authors:** Nouf A Alhammadi, Reema M Bedywi, Rammas A Shawkhan, Adhwaa A Aljari, Sara A Asiri, Jood A Al Hamdan, Shahd S Al-Hassn, Roaa S Alqahtani

**Affiliations:** 1 Rheumatology, King Khalid University, Abha, SAU; 2 College of Medicine and Surgery, King Khalid University, Abha, SAU

**Keywords:** prevalence, aseer region, saudi arabia, irritable bowel disease, migraine

## Abstract

Background: Limited research has been conducted in Saudi Arabia to investigate the incidence and understanding of migraines and irritable bowel disease (IBS) among the population. This study aimed to quantify the prevalence of migraine and IBS within the Aseer region. Furthermore, it aimed to explore the potential association between migraine and IBS.

Methods: The survey questionnaire was distributed through various social media platforms such as Facebook (Meta Platforms, Inc., Menlo Park, California, United States), Twitter/X (X Corp., San Francisco, California, United States), LinkedIn (Microsoft Corporation, Sunnyvale, California, United States), and WhatsApp (Meta Platforms, Inc.) to maximize the reach and engagement of potential participants. The Migraine Screen Questionnaire (MS-Q) assessed the type and frequency of headache pain, along with intensity and impact on daily activities. The questionnaire also incorporated the Rome IV diagnostic criteria for IBS.

Results: A total of 683 participants were included in this study; 65.2% were aged 21-39 years, 85.5% were females, 61.6% were single, and 73.1% had a university degree or higher. Of the participants, 45.97% experienced migraines, while 39.97% exhibited symptoms of IBS. There was a statistically significant association between having IBS and migraine (χ2 = 11.88, p 0.001). Migraine was significantly associated with female sex (p = 0.049), history of psychiatric disease (p < 0.001), and family history of migraines (p < 0.001). IBS was significantly associated with age (p = 0.042), history of psychiatric disease (p = 0.015), and sleeping hours (p = 0.038).

Conclusions: This study reveals a high prevalence of migraine and IBS, underlining their interconnection. Key risk factors include age, gender, family history, and psychiatric disorders. Targeting high-risk groups is crucial due to the substantial impact on daily life and performance.

## Introduction

Migraine constitutes a hereditary, episodic, and intricate disruption in sensory processing, marked by a cluster of symptoms, with headaches being the defining feature [1]. Migraine is the most prevalent neurological problem encountered in Primary Care [2]. As per the results from the latest Global Burden Disease study, migraine maintains its position as the second leading contributor to global disability and takes the first spot among young females [3]. This condition is quite common, affecting 18% of females and 6% of males. In addition, chronic migraine, which impacts 2% of the world's population, poses a substantial burden on individuals, their families, and society at large [4].

Migraine episodes can last 4-72 hours and involve four stages: preliminary phase, aura, headache, and postdrome. The preliminary phase involves non-painful symptoms like yawning, mood shifts, and difficulty concentrating. Aura, a transient neurological manifestation, is common among women. Headache, triggered by trigeminal sensory pathways, causes pulsating pain and disrupts daily activities. Postdrome symptoms include fatigue, drowsiness, concentration difficulties, and increased noise sensitivity [5-8].

Limited research has been conducted in Saudi Arabia to investigate the incidence and understanding of migraines among the local population. Studies reveal a varying prevalence of migraine in different regions. In the Thuqbah neighborhood of Khobar city, the prevalence was found to be 5% [9]. Moving to Aseer in the southern part of the country, the prevalence was higher, reaching 12.3% [10]. In particular, the prevalence was remarkably elevated in Taif, located in the western part of Saudi Arabia, where a substantial 78.5% of the sample experienced migraine headaches. This was a cross-sectional study conducted on 354 individuals using the International Headache Society (IHS) criteria [11]. Finally, in the eastern province, approximately 40% of study participants reported experiencing migraine headaches [12]. In a recent comprehensive study conducted across various regions of the kingdom, the prevalence of migraine was determined to be 27.4% [13]. Indeed, the observed variability in migraine prevalence in different regions in Saudi Arabia could potentially be attributed to the use of distinct assessment methodologies.

IBS is a persistent condition affecting the GI tract, marked by recurring abdominal pain and changes in bowel patterns [14]. Among gastroenterologists, IBS is the most prevalent disorder encountered [15]. Diagnosis relies on the symptom-based classification system known as the Rome Criteria, with the latest iteration, Rome IV, being recently introduced [16]. Calculating incidence rates for IBS is rare, and prevalence figures tend to vary globally, both in different countries and within them. These inconsistencies can be traced back to the diverse nature of prevalence studies, encompassing differences in measurement tools, methodologies, diagnostic criteria, populations under scrutiny, and cultural factors examined [14].

Both disorders are diagnosed based on symptoms. Migraine and IBS have numerous similarities in terms of high frequency, female predominance, chronic and recurring symptoms, etiology, and burden on societal and financial costs [17]. Because numerous illnesses have been linked to the central nervous system and the enteric nervous system, it is considered that the brain-gut axis has a significant impact on how neuronal disorders influence the GI tract [18]. Furthermore, serotonin, cerebral and visceral hypersensitivity, and genetic variables are thought to be common pathogenesis routes for migraine and IBS [17,19].

On a global scale, multiple studies have demonstrated a notable association between migraine and IBS [20,21]. The hypothesis being tested posits a direct link between IBS and migraine, implying that the likelihood of experiencing IBS is heightened among individuals with migraine conditions. The study aimed to assess the prevalence of migraine and IBS within the Aseer region, Saudi Arabia. Furthermore, it aims to explore the potential association between them.

## Materials and methods

A cross-sectional study design was employed for this research in the Asser region of Saudi Arabia. Aseer, located in the southwestern region of Saudi Arabia, constitutes one of the administrative divisions of the country. Its geographical coordinates are defined by a latitude range of 17.25°-19.50° N and a longitude range of 50.00° to 41.50° E. The city of Abha serves as the central hub of this emirate. Encompassing a vast area of 81,000 km², Aseer is home to an estimated population of approximately 1,563,000 residents. The study was conducted from 5 September 2022 to October 10, 2022.

Study population

The study encompassed the complete population living within the Aseer region, Saudi Arabia, that was available during the study timeline. Inclusion criteria applied were: residency in the Aseer region for at least six months, age of 18 years or older, demonstrating the capacity to autonomously communicate and successfully complete the questionnaire, and expressing a willingness to take part in the study.

Sample size and sampling technique

To detect an estimated difference of 20% in the occurrence of migraines between individuals with and without IBS, with a confidence level of 95% and a study power of 80%, a sample size of 450 participants was deemed necessary [13]. The precision was set at 4%. The sample size calculation was performed using the PASS (Process Automation Software System) software for sample size determination. Participants were enrolled using a combination of convenience and snowball sampling techniques. The survey questionnaire was distributed through various social media platforms such as Facebook (Meta Platforms, Inc., Menlo Park, California, United States), Twitter/X (X Corp., San Francisco, California, United States), LinkedIn (Microsoft Corporation, Sunnyvale, California, United States), and WhatsApp (Meta Platforms, Inc.) to maximize the reach and engagement of potential participants.

Data collection

The questionnaire comprehensively captured various aspects related to sociodemographic, medical, and personal habits. The first section focuses on sociodemographic data, including age, gender, marital status, education, occupation, and family income. The subsequent section delved into medical and family history, specifically asking for a history of psychiatric disease and a family history of chronic headache or migraine. The section on personal habits explored smoking habits, intake of energy drinks and soft drinks, and daily sleeping hours. The questionnaire also incorporated the Rome IV diagnostic criteria for IBS, addressing symptoms such as abdominal distension/pain, pain related to defecation, pain associated with changes in stool frequency and appearance, as well as the use of tests for IBS diagnosis.

Additionally, the migraine screening questionnaire (MS-Q) assessed the type and frequency of headache pain, as well as its intensity and impact on daily activities. The questionnaire comprises five questions addressing the frequency, attributes of headaches, and the presence of symptoms associated with migraines. Responses indicating a negative answer (NO) were assigned a score of 0, while positive answers (YES) were scored as 1. A threshold of ≥ 4 points was identified as indicative of potential migraine suspicion, whereas a score < 4 indicated the absence of suspicion for migraines [22].

Ethics

Ethical clearance for the study was obtained from the Research Ethics Committee of Khalid Faculty University, Abha, Saudi Arabia (approval number: ECM#2022-2504). All participants received detailed information about the study and its objectives and they were given the freedom to decide whether to provide their informed consent and participate or decline to participate in the study. The research team placed a strong emphasis on preserving the confidentiality and anonymity of the personal data throughout the research journey.

Statistical analysis

Statistical analysis was performed using the R software version 4.1.1 (R Core Team 2021, R Foundation for Statistical Computing, Vienna, Austria). Categorical variables were presented as frequencies and percentages. The comparison of two independent categorical variables was performed using Pearson's chi-square test. The degrees of freedom associated with the chi-square value indicated the number of independent categories contributing to the analysis. The strength of the association between categorical variables was evaluated using Cramér's V test. This test was applied to categorical variables with more than two levels, producing values ranging from 0 to 1, where 0 represents no association and 1 denotes a perfect association. A p-value below 0.05 was considered statistically significant for this analysis.

## Results

Table 1 presents the demographic characteristics and relevant factors of the study participants (N=683) in relation to pain from the IBS. The age distribution showed that a significant proportion fell within the category of 21-39 years (65.2%), followed by 4-59 years (14.9%) and less than 20 years old (19.2%). The majority of participants were females (85.5%). Almost three-fifths of the participants were single (61.6%), followed by those who were married (36.0%), and a smaller percentage were divorced/widowed (2.3%). In terms of education, almost three-quarters had a university degree or higher (73.1%), with 53.3% being students. Occupation-wise, a notable percentage worked in the government sector (16.1%), while others were employed in the private sector (6.9%), retired (23.7%), or had no job (23.7%). Family income ranged from less than 5,000 Saudi Riyal (SAR) to more than 20,000 SAR, with varying proportions within each category. Furthermore, a small percentage had a medical history of mental illness (15.8%), and a significant proportion reported a family medical history of migraine (33.5%). The majority of participants were non-smokers (87.3%), with a smaller percentage being current (8.3%) or former smokers (4.4%). In terms of daily sleep hours, most reported having six to eight hours of sleep (57.7%), followed by less than six hours (29.6%) and more than eight hours (12.7%).

**Table 1:** Demographic Characteristics and Relevant Factors of Study Participants (N=683) IBS: irritable bowel disease; SAR: Saudi Riyal

Studied variables	Categories of response	n (%)
Do you suffer from colon syndrome pain	Yes, I have IBS and I do not suffer from chronic neurological disease or inflammatory bowel disease.	683 (100%)
	Less than 20 years old	131 (19.2%)
Age in years:	20-39 years	445 (65.2%)
	40-59 years	102 (14.9%)
	60 years and above	5 (0.7%)
Sex:	Female	584 (85.5%)
	Male	99 (14.5%)
Marital Status:	Single/single	421 (61.6%)
	Married	246 (36.0%)
	Divorced/widowed	16 (2.3%)
Education:	Less than secondary	24 (3.5%)
	Secondary education	160 (23.4%)
	University and above	499 (73.1%)
	Student	364 (53.3%)
Occupation:	Work in the government sector	110 (16.1%)
	Work in the private sector	47 (6.9%)
	No job/retired	162 (23.7%)
	Less than 5000 SAR	115 (16.8%)
Family income:	More than 20,000 SAR	118 (17.3%)
	Between 10,000-20,000 SAR	261 (38.2%)
	Between SAR 5,000-9,000	189 (27.7%)
Is there a medical history of mental illness?		108 (15.8%)
Is there a family medical history of the migraine?		229 (33.5%)
Smoking (cigarette/shisha)	Non-smoker	596 (87.3%)
	Current smoker	57 (8.3%)
	Former smoker	30 (4.4%)
Daily sleep hours	6-8 hours	394 (57.7%)
	Less than 6 hours	202 (29.6%)
	More than 8 hours	87 (12.7%)

The distribution of migraine types reveals cluster (11.1%), dull (23.1%), generalized (37.2%), and throbbing (28.6%). Nausea's association with migraines varies, with 14.5% leading to vomiting, 47.9% being mild, and 37.6% showing no nausea. Tolerability levels range from bearable (51.0%) to hardly bearable (38.2%), with 10.8% finding them unbearable, resulting in some being confined to bed (9.7%). Migraine disability spans marked (37.8%), negligible (34.8%), and nil (17.7%). Severity is distributed as mild (19.5%), moderate (50.2%), severe (24.5%), and very severe (5.9%), with 8.1% experiencing daily migraines and 49.3% occurring two to four times a month. Duration varies, with 34.8% experiencing migraines two to four times a week and 7.8% more than once a day (Table 2).

**Table 2:** Characteristics of Migraines: Distribution and Patterns Among Study Participants (N=683)

Measured variables	Characteristics, n (%)	
Type	Cluster	76 (11.1%)
	Dull	158 (23.1%)
	Generalized	254 (37.2%)
	Throbbing	195 (28.6%)
Nausea	Leading to vomiting	99 (14.5%)
	Mild	327 (47.9%)
	None	257 (37.6%)
Tolerability	Bearable	348 (51.0%)
	Hardly bearable	261 (38.2%)
	Unbearable	74 (10.8%)
	Confined to bed	66 (9.7%)
Disability	Marked	258 (37.8%)
	Negligible	238 (34.8%)
	Nil	121 (17.7%)
Severity	Mild	133 (19.5%)
	Moderate	343 (50.2%)
	Severe	167 (24.5%)
	Very severe	40 (5.9%)
	From 2- 4 times/month	337 (49.3%)
Duration	from 2- 4 times/week	238 (34.8%)
	Daily	55 (8.1%)
	More than once/day	53 (7.8%)

**Figure 1:**
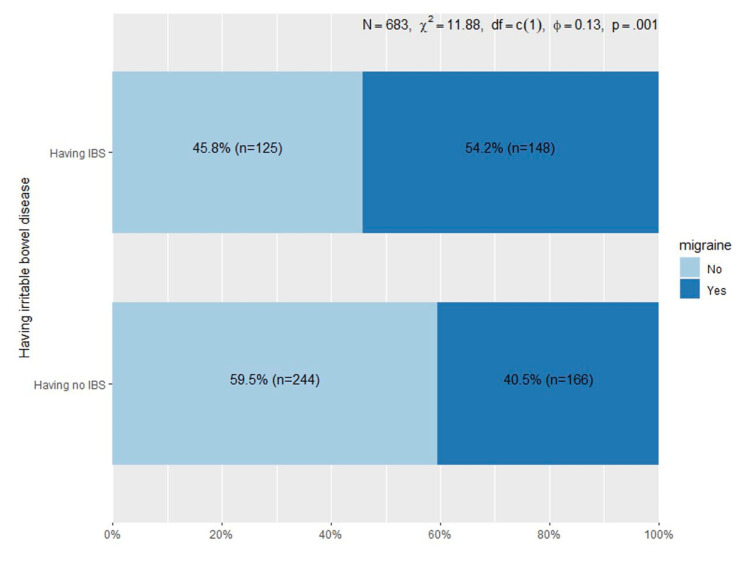
Association Between Migraine and IBS IBS: irritable bowel disease

The results revealed that 45.97% of the participants experienced migraines, while 39.97% exhibited symptoms of IBS. Additionally, a noteworthy correlation was identified between the co-occurrence of these two conditions. There was a statistically significant association between having IBS and migraine (χ2 = 11.88, p 0.001) (Figure 1).

The distribution of the age groups did not indicate a significant difference between individuals with and without migraines (p = 0.537). However, the gender distribution demonstrated statistical significance (p = 0.049), implying a higher proportion of women among those with migraines. Marital status did not exhibit substantial differences (p = 0.174). Similarly, the distribution of education levels (p = 0.906) and the occupational distribution (p = 0.767) did not reveal significant variations between the groups. On the other hand, the history of psychiatric disease significantly differed between the groups (p < 0.001), with a greater proportion of migraine sufferers having a history of psychiatric issues. The presence of a family history of migraines was also significantly related to the occurrence of migraines (p < 0.001). Smoking habits, sleeping hours, and carbonated beverage consumption did not show significant differences (p = 0.875, p = 0.507, p = 0.056, respectively) (Table 3).

**Table 3:** Association of Demographic and Lifestyle Factors with Migraine Prevalence SAR: Saudi Riyal

Measured variables	Response categories	No (n=369), n (%)	Yes (n=314), n (%)	p-value
Age	21 - 39 years	235 (63.7%)	210 (66.9%)	0.537
	40 - 59 years	55 (14.9%)	47 (15.0%)	
	Less than 20 years	75 (20.3%)	56 (17.8%)	
	Above 60 years	4 (1.1%)	1 (0.3%)	
Sex	Female	306 (82.9%)	278 (88.5%)	0.049
	Male	63 (17.1%)	36 (11.5%)	
Marital status	Married	136 (36.9%)	110 (35%)	0.174
	Single	228 (61.8%)	193 (61.5%)	
	Widowed	5 (1.4%)	11 (3.5%)	
Education	Less secondary	13 (3.5%)	11 (3.5%)	0.906
	Secondary	84 (22.8%)	76 (24.2%)	
	University &postgraduate	272 (73.7%)	227 (72.3%)	
Occupation	Government	61 (16.5%)	49 (15.6%)	0.767
	Private sector	22 (6%)	25 (8%)	
	Retired	87 (23.6%)	75 (23.9%)	
	Student	199 (53.9%)	165 (52.5%)	
Income	Above 2000 SAR	67 (18.2%)	51 (16.2%)	0.100
	From 1000 - 2000 SAR	147 (39.8%)	114 (36.3%)	
	From 5000 - 10000 SAR	105 (28.5%)	84 (26.8%)	
	Less than 5000 SAR	50 (13.6%)	65 (20.7%)	
History of psychiatric disease	No	330 (89.4%)	245 (78.0%)	<0.001
	Yes	39 (10.6%)	69 (22.0%)	
Family history of migraine	No	283 (76.7%)	171 (54.5%)	<0.001
	Yes	86 (23.3%)	143 (45.5%)	
Cigarette	Ex-smoker	15 (4.1%)	15 (4.8%)	0.875
	Non smoker	324 (87.8%)	272 (86.6%)	
	Smoker	30 (8.1%)	27 (8.6%)	
Sleeping hours	More than 8 hours	49 (13.3%)	37 (11.8%)	0.507
	From 6 - 8 hours	217 (58.8%)	177 (56.4%)	
	Less than 6 hours	103 (27.9%)	100 (31.8%)	
Carbonated beverage	Always	67 (18.2%)	80 (25.5%)	0.056
	Never	58 (15.7%)	40 (12.7%)	
	Sometimes	244 (66.1%)	194 (61.8%)	

The distribution of age groups appeared to have a statistically significant association with IBS (p = 0.042). Those within the 21-39 age range appear to have a higher prevalence of IBS compared to other groups. There was no significant difference in IBS prevalence based on gender (p = 0.383), marital status (p = 0.777), education (p = 0.185), occupation (p = 0.147), and income (p = 0.071). A history of psychiatric disease had a significant association with IBS prevalence (p = 0.015), as those with a history of psychiatric issues seem to have a higher prevalence of IBS. On the other hand, the presence of a family history of migraines didn't show a statistically significant association with IBS prevalence (p = 0.099). Smoking habits did not significantly influence the prevalence of IBS (p = 0.279). The distribution of sleeping hours showed a statistically significant connection with the prevalence of IBS (p = 0.038). Those with six to eight hours of sleep appeared to have a higher prevalence of IBS. The consumption of carbonated beverages didn't seem to significantly impact IBS prevalence (p = 0.384) (Table 4).

**Table 4:** Association of Demographic and Lifestyle Factors with IBS Prevalence IBS: irritable bowel disease; SAR: Saudi Riyal

Measured variables and responses		No IBS, n (%)	IBS, n (%)	p-value
	Less than 20 years	90 (22.0%)	41 (15.0%)	
Age	21 - 39 years	250 (61%)	195 (71.4%)	0.042
	40 - 59 years	67 (16.3%)	35 (12.8%)	
	Above 60 years	3 (0.7%)	2 (0.7%)	
Sex	Female	355 (86.6%)	229 (83.9%)	0.383
	Male	55 (13.4%)	44 (16.1%)	
Marital status	Married	144 (35.1%)	102 (37.4%)	0.777
	Single	257 (62.7%)	164 (60.1%)	
	Widowed	9 (2.2%)	7 (2.6%)	
Education	Less secondary	14 (3.4%)	10 (3.7%)	0.185
	Secondary	106 (25.9%)	54 (19.8%)	
	University & postgraduate	290 (70.7%)	209 (76.6%)	
Occupation	Government	58 (14.1%)	52 (19%)	0.147
	Private sector	24 (5.9%)	23 (8.4%)	
	Retired	99 (24.1%)	63 (23.1%)	
	Student	229 (55.9%)	135 (49.5%)	
Income	Above 2000 SAR	83 (20.2%)	35 (12.8%)	0.071
	From 1000 - 2000 SAR	155 (37.8%)	106 (38.8%)	
	From 5000 - 10000 SAR	109 (26.6%)	80 (29.3%)	
	Less than 5000 SAR	63 (15.4%)	52 (19%)	
History of psychiatric disease	No	357 (87.1%)	218 (79.9%)	0.015
	Yes	53 (12.9%)	55 (20.1%)	
Family history of migraine	No	283 (69%)	171 (62.6%)	0.099
	Yes	127 (31%)	102 (37.4%)	
Cigarette	Ex-smoker	21 (5.1%)	9 (3.3%)	0.279
	Non smoker	359 (87.6%)	237 (86.8%)	
	Smoker	30 (7.3%)	27 (9.9%)	
Sleeping hours	From 6 - 8 hours	251 (61.2%)	143 (52.4%)	0.038
	Less than 6 hours	116 (28.3%)	87 (31.9%)	
	More than 8 hours	43 (10.5%)	43 (15.8%)	
Carbonated beverage	Always	83 (20.2%)	64 (23.4%)	0.384
	Never	64 (15.6%)	34 (12.5%)	
	Sometimes	263 (64.1%)	175 (64.1)	

## Discussion

This cross-sectional study sought to investigate the occurrence of migraine and IBS in the population residing in the Asser region of Saudi Arabia. The findings revealed that migraine was prevalent in 45.97% of the population, whereas IBS was present in 39.97%. Moreover, a noteworthy correlation was identified between the co-occurrence of these two conditions. We found a significant association between migraine and gender, history of psychiatric diseases and family history of migraine. We also observed a significant association between IBS and age, history of psychiatric diseases, and sleep hours.

In this study, the prevalence of migraine was found to exceed the rates observed in earlier studies [9-11,13]. The heightened occurrence of migraine in the Asser region might potentially be attributed to the area's elevated altitude, resulting in lower oxygen concentrations. There is substantiating evidence suggesting that migraine episodes, particularly those accompanied by aura, could be instigated by a decrease in blood oxygen saturation [23]. A phenomenon referred to as high-altitude headache frequently manifests when ascending beyond 2500 meters above sea level, often exhibiting characteristics reminiscent of migraines. Furthermore, it has been documented that those migraines triggered by sleep apnea display improvement following bariatric surgery, as highlighted in Kallweit et al.'s 2011 study [24]. The predominant types of migraine were generalized and throbbing. The occurrence varied, with it being mild or absent in approximately 90% of the participants. Almost 40% reported severe pain or confinement to bed. The level of migraine-induced disability was significant in around 40% of the respondents, with severe symptoms noted in nearly 25%, and daily or more frequent occurrence in approximately 16%. These findings help to elucidate the burden within the population residing in the Asser region.

Interestingly, we observed a high prevalence of IBS among the studied population. This prevalence is higher than what was derived from a meta-analysis conducted by Oka et al. [25]. In their study, the pooled prevalence of IBS across 53 studies utilizing the Rome III criteria was 9.2% (95%CI 7.6-10.8). The pooled IBS prevalence based on six studies using Rome IV criteria was 3.8% (95%CI 3.1-4.5).

Our findings revealed a noteworthy association between individuals with IBS and migraine complaints. Among respondents who had IBS, 54.2% reported experiencing migraines, while 40.5% of those without IBS indicated migraine complaints. This association demonstrated statistical significance, underscoring a potential link between the two conditions. Kim et al. found that the prevalence of migraine in individuals with GI diseases was found to be 3.46 times higher than in those without GI diseases [26]. Furthermore, upon analyzing specific GI diseases, patients with gastroesophageal diseases, dyspepsia, IBS, and peptic ulcer disease (PUD) (excluding inflammatory bowel disease) exhibited a significantly elevated prevalence of migraines compared to individuals without each respective GI condition. In fact, GI diseases and migraines share intricate yet interconnected underlying mechanisms. Accumulating evidence from various GI diseases indicates that nerve fibers can become hypersensitive to pain due to nerve signals, endocrine influences, or immune responses [27].

We identified a noteworthy correlation between gender and migraine. However, we did not find such an association with IBS. The prevalence of migraines was markedly higher among females compared to males. Epidemiological evidence demonstrates a greater incidence of the condition in females, starting from puberty and persisting throughout life. The gender-based distinctions in migraines extend to clinical significance. Females often experience more frequent, longer-lasting, and more threatening attacks compared to males [28]. This observation has been extensively documented in the literature [29,30]. In the current study, age was significantly associated with IBS while it was not associated with migraine, with the middle age group being more affected than older adults. Numerous studies were in alignment with our findings [31,32].

We found that a family history of psychiatric diseases exhibited a significant association with both migraines and IBS, whereas a family history of migraines was linked to an increased likelihood of experiencing migraines. Migraine, a prevalent and potentially debilitating neurovascular disorder, has demonstrated connections with diverse medical conditions including a range of psychiatric disorders such as depression and anxiety [33]. Research has also highlighted its association with stress-related disorders, including those related to abuse and post-traumatic stress disorder (PTSD) [34]. The appearance of psychiatric comorbidities alongside migraines can be attributed to various possibilities. One theory suggests that psychiatric factors might be a less frequent cause of headaches. On the contrary, it is plausible that the ongoing mental strain from persistent or severe migraines could trigger or contribute to various psychiatric dysfunctions. The most comprehensive theory postulates that both disorders stem from shared pathophysiological irregularities. These could encompass genetic factors or abnormalities in serotonergic processing and responses to estrogen, establishing a common ground for both conditions [34,35]. GI symptoms have been recognized to manifest in response to fear, anxiety, and stress. The dynamics of gut motility have been observed to react to shifts in emotional states. Additionally, stress has been shown to impact both motility and visceral hypersensitivity. In recent times, substantial research has focused on examining the correlation between IBS and psychiatric disorders. The prevalence of co-morbidity with psychiatric conditions ranges from 54% to 94% in patients with IBS who seek treatment [36,37].

Strengths and limitations

This study holds significant significance as it focuses on a specific region of Saudi Arabia, particularly the southern region. However, it's important to acknowledge the study's limitations. The utilization of an online survey introduces the potential for selection bias, which could restrict the representation of the broader community under study. Furthermore, the questionnaire-based survey has the restriction of being unable to exclude alternative migraine and IBS differential diagnoses. In the absence of a complete history, clinical examination, and investigations, other diagnoses may be considered. This is significant since both migraine and IBS are essentially clinical diagnoses after all other possibilities have been ruled out. Additionally, the lack of a random sampling method may impede the applicability of the findings to a larger population. The cross-sectional survey design further limits the ability to establish a cause-and-effect relationship between the identified factors and the occurrence of IBS or migraines. Despite these constraints, the study offers valuable insights into the prevalence and associated factors of IBS and migraines among the Saudi population, laying the groundwork for future research and interventions in this domain.

## Conclusions

This study brings to light a noteworthy portion of the surveyed populace expressing concerns about migraines and IBS. The results underscore a marked correlation between these two conditions. Furthermore, various risk factors have been pinpointed, encompassing middle age, female gender, familial history of migraines, and psychiatric disorders, all of which heighten vulnerability to these conditions. There is a growing imperative to concentrate efforts on high-risk groups, given that these conditions profoundly affect their daily lives and performance, impacting a considerable portion of the population.
